# Regulation of Innate Immune Responses by Bovine Herpesvirus 1 and Infected Cell Protein 0 (bICP0)

**DOI:** 10.3390/v1020255

**Published:** 2009-09-07

**Authors:** Clinton Jones

**Affiliations:** Department of Veterinary and Biomedical Sciences, Nebraska Center for Virology, University of Nebraska, Lincoln, Fair Street at East Campus Loop, Lincoln, NE, 68583-0905, USA; E-mail: cjones@unlnotes.unl.edu; Tel.: +1 (402) 472-1890; Fax: +1 (402) 472-9690

**Keywords:** bovine herpesvirus 1 (BoHV-1), bICP0, interferon, IRF3, IRF7, transcriptional regulation

## Abstract

Bovine herpesvirus 1 (BoHV-1) infected cell protein 0 (bICP0) is an important transcriptional regulatory protein that stimulates productive infection. In transient transfection assays, bICP0 also inhibits interferon dependent transcription. bICP0 can induce degradation of interferon stimulatory factor 3 (IRF3), a cellular transcription factor that is crucial for activating beta interferon (IFN-β) promoter activity. Recent studies also concluded that interactions between bICP0 and IRF7 inhibit trans-activation of IFN-β promoter activity. The C3HC4 zinc RING (really important new gene) finger located near the amino terminus of bICP0 is important for all known functions of bICP0. A recombinant virus that contains a single amino acid change in a well conserved cysteine residue of the C3HC4 zinc RING finger of bICP0 grows poorly in cultured cells, and does not reactivate from latency in cattle confirming that the C3HC4 zinc RING finger is crucial for viral growth and pathogenesis. A bICP0 deletion mutant does not induce plaques in permissive cells, but induces autophagy in a cell type dependent manner. In summary, the ability of bICP0 to stimulate productive infection, and repress IFN dependent transcription plays a crucial role in the BoHV-1 infection cycle.

## 1. Introduction

BoHV-1 infection can cause conjunctivitis, pneumonia, genital disorders, abortions, and an upper respiratory infection known as bovine respiratory disease (BRD) or “Shipping Fever” [136]. BoHV-1 initiates BRD by immunosuppressing cattle [24,48–50,152], which can lead to pneumonia as a result of secondary bacterial infections. BoHV-1 induced immune-suppression can result in secondary bacterial infections (Pasteurella haemolytica, Pasteurella multocida, and Haemophilus somnus for example) that cause life-threatening pneumonia [152]. BRDC and BoHV-1 infections costs the cattle industry at least $3 billion/year in the United States [1,15,24,48–50,71,79,122,136]. Modified live vaccines are available, and in general, they prevent clinical disease in adults. However, the same vaccine strains are immunosuppressive and can cause serious disease in young calves or abortions in pregnant cows.

Like other α-herpesvirinae subfamily members, BoHV-1 establishes lifelong latency in ganglionic neurons of the peripheral nervous system following acute replication in mucosal epithelium [146]. Virus reactivation and spread to other susceptible cattle occur after stress or corticosteroid treatment, which resembles stress [124,134]. There have been increases in BoHV-1 outbreaks in vaccinated feedlot cattle, which are the result of vaccine strains reactivating from latency [33,66,137,139]. The viral protein, bICP0, is crucial for productive infection and reactivation from latency. The bICP0 protein has two known functions that are important for stimulating productive infection and promoting viral pathogenesis: stimulating the activity of all viral promoters and inhibiting interferon dependent transcription. These bICP0 functions are the focus of this review.

## 2. Results and Discussion

### 2.1. bICP0 is encoded within immediate early transcript unit 1 (IEtu1)

Infection of permissive cells [28] or calves [145] leads to rapid cell death, in part due to apoptosis. Viral gene expression is temporally regulated in 3 distinct phases: immediate early (IE), early (E), or late (L). IE gene expression is stimulated by a virion component, bTIF, which interacts with a cellular transcription factor (Oct-1). The bTIF/Oct-1 complex binds to IE promoters and stimulates transcription [49].

**Figure 1.**
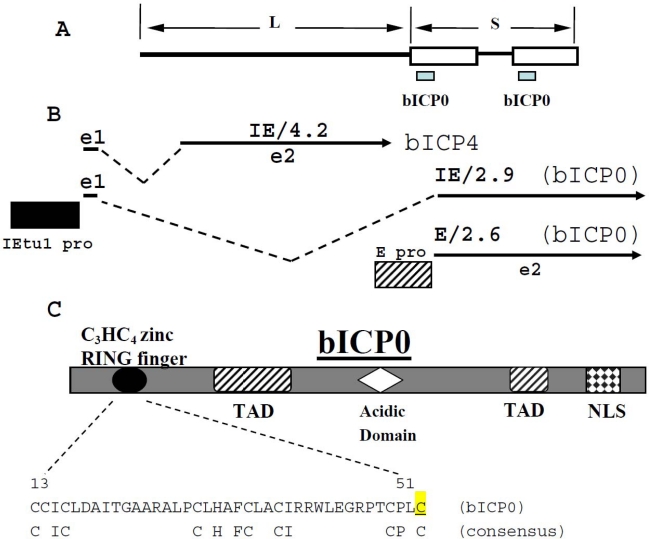
Schematic of bICP0 gene within the BoHV-1 genome. **Panel A:** Schematic of BoHV-1 genome and location of bICP0 gene. The Unique Long (L) and Unique Short (S) regions of BoHV-1 are denoted. **Panel B:** Position of bICP4 and bICP0 transcripts is shown. The immediate early transcription unit 1 (IEtu1) encodes bICP4 (IE/4.2) and bICP0 (IE/2.9) [148,149]. The IEtu1 promoter activates IE expression of IE/4.2 and IE/2.9 (IEtu1 pro). E/2.6 is the early transcript that encodes bICP0 and an early promoter (E pro) activates expression of this transcript [147]. Exon 2 (e2) of bICP0 contains all of the protein coding sequences of bICP0. The dashed lines are intron sequences. **Panel C:** Schematic of bICP0 protein and known functional domains. The functional domains include a NLS (nuclear localization sequence, TAD (transcriptional activation domain), Acidic Domain, and the C_3_HC_4_ zinc RING finger. Amino acid sequence (residue 13–51) of the C_3_HC_4_ zinc RING finger in bICP0 is shown. The consensus residues in a C_3_HC_4_ zinc RING finger are also shown. The mutated cysteine at position 51 is highlighted in yellow and is underlined.

Two IE transcription units exist: IEtu1 and IEtu2. IEtu1 encodes homologues of two human herpesvirus-1 (HHV-1) proteins, ICP0 and ICP4. IEtu2 encodes a protein similar to the HSV ICP22 protein [69]. IE proteins activate E gene expression, and viral DNA replication occurs. L gene expression then occurs, culminating in virion assembly and release. bICP0 is crucial for productive infection because it activates all viral promoters [43,147,149]. Relative to other alpha-herpesvirus members, the organization of the BoHV-1 ICP4 and ICP0 genes is unique because a common IE promoter (IEtu1) drives expression of bICP0 and bICP4 [147] (Figure 1B). bICP0 also contains an E promoter located near the 5′ end of the coding exon of bICP0, which is positively regulated by bICP0. Expression of bICP4 leads to repression of IEtu1 promoter activity. Thus bICP0 is considered to be the major viral regulatory protein that stimulates productive infection [43,147–149]. The bICP0 E promoter is activated by dexamethasone [150], a stimulus that induces reactivation from latency suggesting the E promoter plays a role in the early phases of reactivation from latency [75,76].

### 2.2. bICP0 contains multiple functional domains

bICP0 [70], HHV-1 encoded ICP0 [34–37,93], and equine herpesvirus 1 encoded ICP0 [16,17] contain a C_3_HC_4_ zinc RING finger near their N terminus that activates productive infection (Figure 1C). ICP0 [38–40,102,103] and bICP0 [70,114] localize with and disrupt promyelocytic leukemia (PML) protein-containing nuclear domains. The C_3_HC_4_ zinc RING finger domains located in bICP0 [29] and ICP0 [13,14,140] possess intrinsic E3 ubiquitin ligase activity. Thus, bICP0 and ICP0 can induce ubiquitin dependent proteolysis of specific proteins [38,40,85,115]. The specific proteins that are targets for proteolysis by bICP0 have not been identified.

bICP0 contains two transcriptional activation domains (TAD) that were identified by transposon insertion studies [157] (Figure 1C). A nuclear localization signal (NLS) is also located near the C-terminus of bICP0 and deletion of sequences encompassing the NLS impairs bICP0 function [128,129,157]. An acidic domain, which can be found in many transcriptional activators, is adjacent to the C_3_HC_4_ zinc RING finger. To date, the acidic domain does not appear to play a role in stimulating transcription or inhibiting IFN-dependent transcription [129,157].

### 2.3. bICP0 interacts with chromatin remodeling enzymes

The ability of bICP0 to interact with chromatin-remodeling enzymes correlates with activating viral transcription. For example, bICP0 interacts with histone deacetylase 1 (HDAC1), and can relieve HDAC1 induced repression of a model promoter [158]. Secondly, bICP0 interacts with p300, a histone acetyl transferase (HAT), and this interaction correlates with activating a late viral promoter [159]. Interestingly, a histone deacetylase inhibitor, trichostatin A, and HHV-1 encoded ICP0 has similar effects on cellular and viral gene expression [62]. Finally, HHV-1 encoded ICP0 interacts with class II HDACs [94]. Furthermore, ICP0 can interfere with histone deacetylation, which correlates with stimulating viral gene expression [52,121].

In general, the eight HDAC family members repress transcription by deacetylating histones [41,51,82,107,141]. HDACs are recruited to specific promoters by interacting with sequence specific DNA binding proteins [10,27,31,57,97,98]. Conversely, HAT family members activate transcription by acetylating histones [83], and non-histone transcription factors [18,25,53,67,101,111,135,156]. These studies suggested that interacts with bICP0 and complexes that contain chromatin-remodeling enzymes play a crucial role with respect to the ability of bICP0 to activate viral transcription.

### 2.4. Activation of the interferon pathway following infection

Infection of bovine cells or calves with BoHV-1 leads to a robust interferon (IFN) response [116]. Relative to HHV-1 or HHV-2, BoHV-1 DNA contains high levels of unmethylated CpG DNA [96], which can trigger the innate immune response via TLR9 [4,55]. Thus, BoHV-1 DNA alone can induce innate immune responses. Although it seems likely that additional viral encoded products can induce innate immune responses, they have not been identified. To give insights into the regulation of BoHV-1, a brief description of how HHV-1 regulates an innate immune response is summarized below.

Infection of cultured human cells with HHV-1 leads to production and secretion of interferon (IFN). The HHV-1 glycoprotein gD induces IFN-α production in mononuclear cells [80], in part, because HHV-1 activates IRF-3 (interferon response factor 3) in certain cell types [123]. Entry of enveloped viruses into cells, including HHV-1 and BoHV-1, also induces innate immune responses that are IFN independent [113]. ICP0, ICP34.5, and Us11 are the known HHV-1 genes that inhibit IFN activation after infection [89,108–110,120]. It seems clear that multiple mechanisms lead to innate immune responses following infection with BoHV-1 and HHV-1.

### 2.5. bICP0 inhibits beta interferon promoter (IFN-β) promoter activity

#### 2.5.a. Activation of the IFN-β promoter is an early event after virus infection

**Figure 2.**
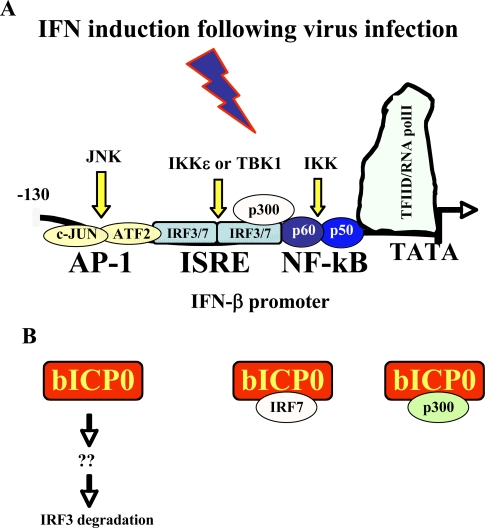
Activation of IFN-β promoter activity and how bICP0 inhibits IFN-β promoter activity. **Panel A:** Schematic of the human IFN-β promoter necessary for inducing an IFN response to virus infection [2,80,86]. The NF-κB binding site is bound by the two proteins, p60 and p50, that comprise the NF-κB transcription factor. A summary of signaling pathways that induces IFN-β promoter activity is presented [7,32,42,54,72,109,119,133,142,143,153]. **Panel B:** A schematic of the known steps by which bICP0 inhibits IFN-β promoter activity. For details, see text.

Activation of the IFN-β promoter is an early response to viral infection [2,80,86] (summarized in Figure 2A). The IFN-β promoter contains three distinct transcription factor binding sites, AP-1 (activating protein 1), the IFN response element (ISRE), and NF-κB. These transcription factor-binding sites are necessary for induction of the IFN response following virus infection. Induction of toll like receptors can activate IRF-3 and the transcription factor NF-κB, and consequently the IFN-β promoter is activated [7,32]. Following virus infection, two cellular protein kinases, IKK-ε (I Kappa kinase epsilon) or TBK-1 (tank binding kinase 1), phosphorylate serine residues at the C-terminus of IRF3 (interferon response factors 3), which induces IRF3 homodimerization and nuclear translocation [42,133]. Nuclear IRF3 associates with other transcriptional activators resulting in direct binding and stimulation of IFN-β promoter activity [142]. IRF3 also directly binds several consensus DNA binding sites, including ISRE (IFN response elements), which stimulates transcription of IFN-stimulated genes in the absence of IFN [54,109].

The serine/threonine protein kinase family (IKK) regulates the activity of NF-κB and to a certain extent JNK (c-jun N-terminus kinases) [119]. JNK1 and JNK2, both serine/threonine protein kinases that are activated by stress can positively activate c-jun, a key component for stimulating AP-1 binding sites [72], including the AP-1 site in the IFN-β promoter. The histone acetylase, p300, is a co-activator for the IFN-β promoter and numerous IFN induced genes [143,153]. Collectively, the activation and association of these transcription factors with the IFN-β promoter is required for the IFN response following virus infection.

#### 2.5.b. bICP0 inhibits IRF3 functions by inducing its degradation

Mice lacking type I and type II interferon receptors in combination with *RAG-2* gene deletions die within a few days following BoHV-1 infection [3]. In contrast, BoHV-1 infection of wt mice does not lead to clinical symptoms or extensive viral replication highlighting the importance that IFN plays in controlling BoHV-1 replication and pathogenesis.

**Figure 3.**
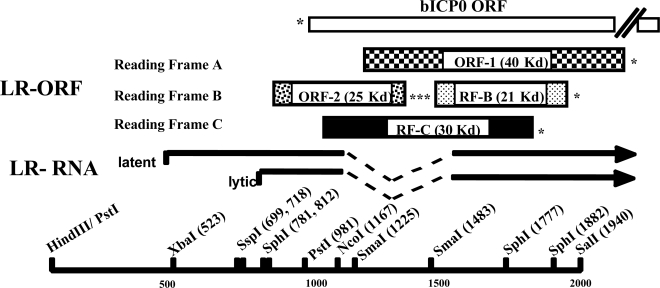
Schematic of the BoHV-1 LR gene and primers used in this study. Partial restriction map, location of LR-RNA, organization of LR ORFs, and the bICP0 ORF. Start sites for LR transcription during latency and productive infection were previously described [44]. Reading Frames B and C contain an open reading frame, but lack an initiating Met. The (*) denotes the positions of stop codons that are in frame with the respective ORF.

In the absence of other viral genes, bICP0 inhibits interferon signaling [59]. bICP0 (directly or indirectly) reduces IRF3 protein levels in human or bovine cells (Figure 3B), which consequently leads to reduced IFN-β promoter activity [127] (Figure 2B). The C_3_HC_4_ zinc RING finger of bICP0 [29,91] is important for inducing IRF3 degradation suggesting bICP0 can ubiquitinate IRF3. The finding that a functional proteasome is necessary for bICP0 induced IRF3 degradation [127] further supports the notion that the bICP0 RING finger mediates IRF3 degradation. However, direct evidence demonstrating that bICP0 ubiquitinates IRF3 is lacking. The C_3_HC_4_ zinc RING finger is not the sole component necessary for bICP0 induced IRF3 degradation because specific mutations near the C-terminus of bICP0 are also important [127].

#### 2.5.c. bICP0 interacts with IRF7

IRF7 was originally identified as a protein that binds and represses the Epstein-Barr virus Qp promoter [155]. IRF7 stimulates IFN-α/β expression [9,100], and thus is an important regulator of the innate immune response [63]. IRF7, like IRF3, undergoes virus-induced activation and phosphorylation at its C-terminus [9,99,130]. Phosphorylation of IRF7 promotes retention in the nucleus, binding to ISRE sequences in promoters of IFN responsive genes, and interactions with IRF3. IRF3 activation is an immediate early regulator of the IFN response, whereas IRF7 is believed to be a component of the early response [131,153,154]. Surprisingly, IRF7 is more important with respect to inhibiting viral infection when IRF3 versus IRF7 knockout mice are compared [63].

Although bICP0 inhibited the ability of IRF7 to stimulate IFN-β promoter activity [127], it was not clear whether bICP0 had a direct effect on IRF7 or if reduced IRF3 levels inhibited the trans-activation potential of IRF7. To test whether bICP0 could inhibit IFN-β promoter activity in the absence of IRF3, undifferentiated embryonic murine teratocarcinoma (P19) cells were utilized. P19 cells, like most undifferentiated teratocarcinoma cell lines, do not express detectable levels of IRF3 or IRF7, and consequently IFN-β promoter activity is not induced following virus infection [151]. However, an IRF7 expression plasmid trans-activated the human IFN-β promoter in P19 cells [128]. The ability of bICP0 to inhibit the ability of IRF7 to trans-activate the IFN-β promoter indicated bICP0 had a direct effect on the functions of IRF7. In contrast to bICP0 inducing reduced levels of IRF3 [127], bICP0 had no effect on IRF7 protein levels in cells transfected with bICP0 or infected cells [127,128]. However, bICP0 interacted with IRF7 or a complex containing IRF7 (Figure 2B), and this interaction correlated with the ability of bICP0 to inhibit the ability of IRF7 to trans-activate IFN-β promoter activity. Finally, the ability of bICP0 to interact with p300 or p300 containing complexes (Figure 2B) may also interfere with IFN-β promoter activity because p300 is crucial for stimulating IFN-β promoter activity.

### 2.6. Growth properties of bICP0 mutant viruses

A bICP0 null mutant virus was generated by homologous recombination [47]. The bICP0 null mutant does not produce plaques; it grows poorly in bovine cells, and does not grow at all in calves. Interestingly, the bICP0 null mutant persists in bovine cells following infection. Additional studies suggested that the bICP0 null mutant induced cytoplasmic vesicles following infection of bovine kidney cells, but not rabbit skin cells [46]. These cytoplasmic vesicles appear to be autophagosomes suggesting that the bICP0 null mutant induced autophagy, a form of programmed cell death [6,11,22]. The bICP0 null mutant, unlike wild type BoHV-1, does not protect infected cells from UV induced apoptosis. The ability of bICP0 to induce viral gene expression appears to be the reason why the bICP0 null mutant does not protect cells from apoptosis because bICP0 is toxic to cells in transient transfection assays [60,70].

A bacterial artificial chromosome (BAC) that contains the Cooper strain of BoHV-1 (pBoHV-1BAC) was used to introduce a single cysteine to glycine mutation into the 51st amino acid of bICP0 (51g mutant), which is within the C_3_HC_4_ zinc RING finger of bICP0 [126]. The 51g mutant grows poorly compared to the rescued virus or the wt BAC derived Cooper strain of BoHV-1. However, the 51g mutant, unlike the bICP0 deletion mutant, induces plaques following infection of cultured bovine cells.

During transient transfection [70,157] or productive infection, higher levels of bICP0 are detected in cells expressing bICP0 proteins containing mutations within the C_3_HC_4_ zinc RING finger. Since it is well established that HHV-1 ICP0 zinc RING finger mutants have increased ½ lives because they cannot induce their own ubiquitination [14,23,34], we tested whether bICP0 regulates its own ½ life by self-ubiquitination. Not surprising, C_3_HC_4_ zinc RING finger of bICP0 [126] regulates the ½ life of bICP0. Furthermore, transient transfection assays and cell free assays demonstrated that the bICP0 protein possess E3 ubiquitin ligase activity, and that the C3HC4 zinc RING finger is crucial for this activity [29].

HHV-1 encoded ICP0 plays a crucial role in allowing virus replication in the face of an IFN response [108–110]. The ICP0 homologue (EP0) encoded by pseudorabies virus is also important for counteracting the antiviral state in swine cells, but not cells from non-natural-host cells [20]. The ability of the 51g mutant virus to grow in bovine cells pretreated with imiquimod is greatly reduced compared to the rescued virus (51gR). Imiquimod stimulates IFN as well as cytokine production [104], and IFN-β promoter activity in bovine cells [116]. Since bICP0 can inhibit IRF3 and IRF7 dependent activation of the IFN-β promoter [127,128], bICP0, directly or indirectly, overcomes the effects of imiquimod. It is tempting to conclude that the ability of bICP0 to inhibit IFN dependent transcription is the reason why the 51g mutant grows poorly in bovine cells after imiquimod treatment. However, the poor growth of the 51g mutant may reflect the fact that disabled viruses do not grow efficiently in suboptimal growth conditions.

In calves, the 51g mutant grows poorly and does not reactivate from latency following treatment with dexamethasone. As expected, the wt BAC derived Cooper strain and the 51g rescued virus reactivated from latency following dexamethasone treatment [92,129]. The current evidence supports a crucial role for wt expression of bICP0 for the growth of BoHV-1 in cultured cells and calves.

### 2.7. Regulation of immune responses by other BoHV-1 genes

#### 2.7.a. Inhibition of CD8+ T cell recognition in infected cells

The BoHV-1 UL49.5 ORF impairs CD8+ T cell recognition of infected cells by repressing expression of the major histocompatibility complex class I and the transporter associated with antigen presentation (TAP) [56,61,112]. UL49.5, also referred to as glycoprotein N (gN), encodes a 96 amino acid protein with an apparent molecular mass of 9 kDa [88] that inhibits TAP (transporter associated antigen processing) functions. The gN proteins encoded by pseudorabies virus and BoHV-1 inhibit TAP-mediated transport of cytosolic peptides into ER, which consequently blocks the assembly of peptide containing ternary MHC-I complexes *in vitro* in virus-infected cells [81,90]. Furthermore, the BoHV-1 UL49.5 protein targets the TAP complex for proteosomal degradation [81]. The UL49.5 protein contains a 22 amino acid signal peptide at its N-terminus, an extracellular domain of 32 amino acids, a transmembrane region of 25 amino acids, and a 17 amino acid cytoplasmic tail [88]. The UL49.5 protein is a non-glycosylated type I membrane protein [87,88].

Peptide transport by TAP is a critical step in MHC class I antigen presentation [5,8,65,81]. In the absence of a functional TAP transporter, most MHC class I molecules are not loaded with peptides [65]. However, they are retained within the ER and ultimately directed for degradation by the proteasome [65]. A gN mutant that lacks the cytoplasmic tail can still bind to the TAP complex and block peptide transport, but this mutant gN protein does not degrade TAP [81]. If the transmembrane domain and cytoplasmic tail are truncated, this gN mutant protein does not block TAP functions [81]. Therefore, sequences within the transmembrane domain of gN appear to be necessary for interacting with TAP. The phenotype of a viral strain expressing the various gN mutant proteins has not been tested in cattle.

#### 2.7.b. Inhibition of CD4+ T cell functions

CD4+ T cell functions are impaired during acute infection of calves, in part, because BoHV-1 infects CD4+ T cells and induces apoptosis [145]. It appears that CD4+ T cells are semi-permissive for BoHV-1 infection suggesting that binding of virus to a receptor or the early events of virus infection induces apoptosis. Unlike CD8 T cell functions that are specifically blocked by gN, it is not clear why CD4+ T cells are very susceptible to apoptosis after infection.

#### 2.7.c. BoHV-1 encodes a protein that interacts with chemokines

Chemokines are small proteins (8–10 kd) that function as cytokines, and thus regulate trafficking and effector functions of leukocytes [12]. As such, chemokines are important regulators of inflammation, immune surveillance, and they have potent anti-viral functions. Functionally, chemokines can be divided into two groups: pro-inflammatory chemokines that are inducible and housekeeping chemokines that are constitutively expressed. Activation of chemokine functions are dependent on selective recognition and activation of chemokine receptors belonging to the seven-membrane domain, G protein-coupled receptor super family. Chemokines can also bind to glycosaminoglycans (GAGS). Chemokine binding to GAGS on cells, in particular endothelial cells, results in chemotactive chemokine gradients that allow the correct presentation of chemokines to leukocytes and therefore enable target cells to cross the endothelial barrier and migrate to tissues.

BoHV-1, BoHV-5, and equine herpesvirus 1 encode a glycoprotein (gG) that is secreted from infected cells, and can bind to a broad range of chemokines [21]. Interactions between gG and chemokines block chemokine activity by preventing their interactions with specific receptors and GAGS. By preventing chemokine-GAG interactions, gG disrupts chemokine gradients, which controls the local environment surrounding an infected cell. A BoHV-1 gG deletion mutant was reported to have reduced virulence [78] suggesting gG is a viral immune evasion gene. However, the exact role of gG in virulence requires additional studies because the gG mutant that was examined was not rescued, and expression of surrounding genes was not examined.

#### 2.7.d. LR RNA can influence immune responses

The latency related (LR) gene is abundantly transcribed in trigeminal ganglia of latently infected calves [84,124,125], and is anti-sense with respect to the bICP0 gene [75–77] (Figure 3A). The LR gene has two open reading frames (ORF-1 and ORF-2), and two reading frames that lack an initiating ATG (RF-B and RF-C) (Figure 3A). A mutant BoHV-1 virus (LR mutant virus) that contains 3 stop codons at the beginning of ORF-2, and lacks 25 bp of wt sequences at the beginning of ORF2 was constructed [69] (Figure 3B). The LR mutant virus grows to similar titers as wt BoHV-1 or the LR rescued virus in cultured bovine cells indicating expression of LR proteins is not necessary for productive infection. When bovine cells are infected with the LR mutant virus, proteins containing ORF-2 or RF-C are not expressed [64,73,74]. Calves infected with the LR mutant virus exhibit diminished clinical symptoms, and reduced shedding of infectious virus in the eye, tonsil, or TG [68,69,118]. The LR mutant virus does not reactivate from latency following dexamethasone treatment [68] indicating LR protein expression is crucial for the latency-reactivation cycle. LR gene products inhibit mammalian cell growth [44,132], bICP0 expression [19,45,132], and apoptosis [26,58]. LR protein expression is necessary for inhibiting apoptosis, in part, because a LR protein binds to two proteins that induce apoptosis, Bid and Cdc42 [105]. LR protein expression is not necessary for inhibiting cell growth or bICP0 expression. It appears that a small regulatory RNA, perhaps a micro-RNA that is encoded by the LR gene inhibits cell growth.

The LR mutant virus induces higher levels of IFN-β3 RNA and to a lesser extent IFN-β2 RNA when compared to wild type BoHV-1 or the LR rescued virus that grows like wild type BoHV-1 [116]. In contrast to humans or mice, cattle contain three different IFN-β genes that are differentially regulated because they have distinct promoters [138,144]. The LR mutant virus prematurely expresses LR-RNA in productively infected cells [116]. Since LR-RNA has the potential to base pair with bICP0 RNA, the formation of double stranded RNA may induce higher levels of IFN-β during the early stages of virus infection. The ability of LR-RNA to induce IFN may promote establishment and maintenance of latency because of the anti-viral effects of IFN.

LR gene products inhibit infiltration of inflammatory cells into trigeminal ganglia of infected calves [117]. It is not clear whether this effect is a direct effect, but a protein encoded by the LR gene can interact with a B-cell and monocyte-activating chemokine precursor [106]. Enhanced infiltration of inflammatory cells into trigeminal ganglia during acute infection correlates with enhanced neuronal death and the inability of the LR mutant virus to reactivate from latency [68,95].

## 3. Conclusions

The ability of viruses to inhibit innate immune responses dictates host range, the pathogenic potential of a virus, and the ability of a given virus to survive in nature. The bICP0 protein has at least two important functions that play an important role during infection of cattle: 1) stimulates viral transcription, and 2) inhibits innate immune responses. bICP0 is a unique viral transcription factor because there is currently no evidence to indicate that it binds specifically (or directly) to DNA. The ability of bICP0 to regulate gene expression and innate immune responses is linked to its ability to interact with cellular regulatory factors. For example, bICP0 can interact with HDAC1, p300, and IRF7 [128,158,159]. The ability of bICP0 to function as an E3 ligase [29,30] correlates with its ability to activate transcription [70,157–159] and inhibit IFN dependent transcription [59,127]. To date, the specific cellular proteins that are targets for proteasomal degradation induced by bICP0 have not been identified. Identification of the cellular proteins that are degraded by bICP0 would greatly enhance our understanding of how bICP0 inhibits IFN dependent transcription and stimulates viral gene expression. In addition to bICP0, BoHV-1 has additional means by which is can regulate cellular immune responses. For example, the ability of BoHV-1 to infect CD4+ T cells and kill these cells would transiently repress immune responses. Furthermore, the ability of UL49.5 to inhibit CD8 T cell recognition of infected cells is an important mechanism by which BoHV-1 evades immune responses. Finally, the viral encoded chemokine, gG, would also appear to play an important role in immune evasion. In conclusion, BoHV-1 has multiple genes that inhibit specific arms of immune responses, which are necessary for growth in cattle.
